# Interplay Between Phosphorylation and O-GlcNAcylation of Sarcomeric Proteins in Ischemic Heart Failure

**DOI:** 10.3389/fendo.2018.00598

**Published:** 2018-10-05

**Authors:** Thomas Mercier, Marion Bouvet, Emilie Dubois-Deruy, Arthur Dechaumes, Olivia Beseme, Vincent Richard, Paul Mulder, Florence Pinet

**Affiliations:** ^1^INSERM U1167 Unité d'Epidémiologie et de Santé Publique, Lille, France; ^2^INSERM UMR1096, Endothélium, Valvulopathies et Insuffisance Cardiaque, Rouen, France

**Keywords:** heart failure, systolic, desmin, interplay, rat models, O-GlcNAcylation, phosphorylation

## Abstract

Post-translational modifications (PTMs) of sarcomeric proteins could participate to left ventricular (LV) remodeling and contractile dysfunction leading in advanced heart failure (HF) with altered ejection fraction. Using an experimental rat model of HF (ligation of left coronary artery) and phosphoproteomic analysis, we identified an increase of desmin phosphorylation and a decrease of desmin O-N-acetylglucosaminylation (O-GlcNAcylation). We aim to characterize interplay between phosphorylation and O-GlcNAcylation for desmin in primary cultures of cardiomyocyte by specific O-GlcNAcase (OGA) inhibition with thiamet G and silencing O-GlcNAc transferase (OGT) and, in perfused heart perfused with thiamet G in sham- and HF-rats. In each model, we found an efficiency of O-GlcNAcylation modulation characterized by the levels of O-GlcNAcylated proteins and OGT expression (for silencing experiments in cells). In perfused heart, we found an improvement of cardiac function under OGA inhibition. But none of the treatments either in *in vitro* or *ex vivo* cardiac models, induced a modulation of desmin, phosphorylated and O-GlcNAcylated desmin expression, despite the presence of O-GlcNAc moities in cardiac desmin. Our data suggests no interplay between phosphorylation and O-GlcNAcylation of desmin in HF post-myocardial infarction. The future requires finding the targets in heart involved in cardiac improvement under thiamet G treatment.

## Introduction

Heart failure (HF) following myocardial infarction (MI) is characterized by alterations of left ventricle (LV) structure and function, known as LV remodeling (1); this pathophysiological process is a strong predictor of both HF and death as we recently showed in two cohorts of patients REVE and REVE2 dedicated to the analysis of LV remodeling (2, 3). The long-term (>10 years) clinical follow-up of patients included in these two cohorts has shown that LV remodeling remains independently associated with HF and cardiovascular death (4).

Evaluation of post-translational modifications (PTM) of sarcomeric cardiac proteins is a promising new approach to studying the mechanisms of HF. The phosphorylation status of sarcomeric proteins is altered in HF and may thus contribute to the decreased cardiac function (5). Another rapid, dynamic, and reversible PTM is O-N-acetylglucosaminylation (O-GlcNAcylation) (6). Both phosphorylation and O-GlcNAcylation regulate numerous cellular functions by reversibly adding either phosphate or O-N-acetylglucosamine (O-GlcNAc) to proteins. The crosstalk between these two PTMs may occur by steric competition for occupancy at the either the same or a proximal amino acid site (7). Specific sites of O-GlcNAcylation described in some cardiac myofilament proteins have suggested that O-GlcNAc and O-phosphate modifications of these proteins may interact dynamically (8, 9).

Recently, we have demonstrated interplay between Ser^208^-phosphorylation and Ser^190^-O-GlcNAcylation of troponin T in ischaemic HF, linked to decreased activity of both PKCε and O-GlcNAcase (OGA) and increased O-GlcNAc transferase (OGT) activity (10). We also showed recently than another sarcomeric protein, desmin has been identified by differential proteomic analysis to have increased levels of phosphorylation in LV of HF-rats compared to the sham-rats (11). In addition, we found a 2-fold increased serine-desmin phosphorylation in the LV of HF-rats, mainly in the insoluble fraction, suggesting the formation of desmin aggregates, toxic for the cardiomyocyte. Desmin is a 53 kDa protein, particularly localized to the Z-band and is considered as a major integrator of contractile apparatus and a critical factor for maintaining intermediate filament structure. Its increased phosphorylation might lead to the network destabilization and formation of aggregates toxic for the cardiomyocyte (12).

Our objectives were to characterize (1) whether the levels of O-GlcNAcylated desmin are regulated in cardiomyocyte by inhibition or activation of O-GlcNAcylation in *in vitro* and *ex vivo* cardiac models of HF and; (2) whether modulation of O-GlcNAcylation impacts the phosphorylation levels of desmin with the aim to decrease the phosphorylation levels of desmin and the formation of desmin aggregates following HF development.

## Materials and methods

### Experimental rat model of ischemic heart failure

All animal experiments were performed according to the Guide for the Care and Use of Laboratory Animals published by the US National Institutes of Health (NIH publication NO1-OD-4-2-139, revised in 2011). Animals were used and experimental protocols performed under the supervision of a person authorized to perform experiments on live animals (F. Pinet: 59-350126). Approval was granted by the institutional ethics review board (CEEA Nord Pas-de-Calais N°242011, January 2012). Before surgery, rats were anesthetized [sodium methohexital, 50 mg/kg intraperitoneal (IP)], while analgesia was administered before (xylazine 5 mg/kg IP) and 1 h after surgery (xylazine 50 mg/kg subcutaneously) as previously described (13). MI was induced in 10-week-old male Wistar rats (Janvier, Le Genest St isle, France) by ligation of the left anterior descending coronary artery according to the method previously described (1, 13). Haemodynamic and echocardiographic measurements were taken at 2 months after surgery, followed by heart excision as previously described (14). Tissues were kept at −80°C until analysis.

### Isolated heart perfusion

*In vitro* LV function was determined in randomly selected control male Wistar rats either untreated (bodyweight: 453 ± 7 g) or treated (bodyweight: 444 ± 14 g) with 100 μL OGA inhibitor, Thiamet G (200 μM diluted in saline buffer, Sigma-Aldrich, Lyon, France) for 2 h. After anesthesia (50 mg/kg of sodium pentobarbital injected intraperitoneally), the heart was rapidly excised and plunged in ice-cold oxygenated KH buffer (5.5 mM glucose, 1.25 mMCaCl_2_, 120 mM NaCl, 31 mM NaHCO_3_, 4.7 mM KCl, 1.2 mM MgSO_4_, 1.2 mM KH2PO4, [pH 7.4]) as previously described (10). The heart was transferred within 30 s to a Langendorf heart perfusion apparatus and perfused at constant hydrostatic pressure (90 mm Hg). A balloon was inserted into the LV and connected to a pressure transducer to record LV (systolic and diastolic) pressure, LV developed pressure and heart rate for 25 min. The balloon was inflated with water, allowing a similar and constant LV distending pressure of 10 mm Hg. At the end of each experiment, the LV was snap-frozen in liquid nitrogen and stored at −80°C until analysis.

### Cell culture

#### Primary cultures of neonatal rat cardiomyocytes

Primary cultures of rat neonatal contractile cardiac myocytes (NCM) were prepared from heart ventricles of 1- or 2-day-old rats as previously described (10). Briefly, cardiac cells of newborn rats' ventricles were dissociated by enzymatic digestion with 0.04% collagenase II (Worthington, Lakewood, NJ, USA) and 0.05% pancreatin (Sigma-Aldrich). Non-NCM were removed by 30 min centrifugation at 1,600 g in a discontinuous Percoll gradient (bottom 58.5%, top 40.5% [v/v], Sigma-Aldrich). NCM were then seeded at a density of 4 × 10^5^ cells per well in 6-well plates coated with 0.01% of collagen (Sigma-Aldrich) (8 × 10^5^ cells per well when they are seeded on coverslip) and cultured in a medium containing DMEM/Medium199 (4:1), 10% horse serum (Life Technologies), 5% fetal bovine serum (FBS) (ATCC), 1% penicillin and streptomycin (10,000 U/mL, Life Technologies) for 7 days at 37°C under 5% CO_2_ atmosphere.

#### Small interfering RNA transfection

The first two individual pre-designed specific siRNA specifically targeting rat OGT mRNA, rat OGT and non-targeting control were used (ON-TARGETplus siRNA, Dharmacon, GE Healthcare). NCMs were plated (100,000 cells/well) in 6-well plates and were allowed to grow for 24 h without antibiotics. The first 2 individual OGT (OGT1 and OGT2) siRNAs (5 nmol/L) were transfected with the DharmaFECT® reagent (4 μL) according to the manufacturer's recommendations. Total cell extracts were collected 72 h after transfection.

### Protein extraction

Proteins from human hearts and rat LVs were extracted from frozen tissues (after removing the infarcted area) with Dounce-Potter homogenization into ice-cold RIPA buffer (50 mM Tris [pH7.4], 150 mM NaCl, 1% Igepal CA-630, 50 mM deoxycholate, and 0.1% SDS) containing antiproteases (Complete™ EDTA-free, Roche Diagnostics), serine/threonine and tyrosine protein phosphatase inhibitors (Phosphatase inhibitor Cocktail 2 and 3, Sigma-Aldrich), 1 mM Na_3_VO_4_ and PUGNAC (50 μM). After 1 h incubation at 4°C, the homogenate was centrifuged at 15,300 g for 15 min at 4°C and the supernatant containing soluble proteins was collected. After treatments, cells were rinsed twice with PBS before being mechanically scraped from the plate in 50 μL of ice-cold RIPA buffer. Soluble and insoluble proteins were extracted as describe above. Protein concentrations for all samples were determined with a Bradford-based method protein assay (Biorad, Marnes-la-Coquette, France).

### Immunoprecipitation, western blot, phos-tag™, and WGA gels

#### Immunoprecipitation

Immunoprecipitation was performed with 50 μg of NCM proteins or 100 μg of LV proteins pre-cleared by incubation with protein A/G magnetic beads (88802, Pierce) for 1 h at 4°C with gentle shaking. The pre-cleared proteins were then mixed with 2 μL of anti-desmin antibody (ab3236, Abcam) diluted in 100 μL of RIPA 1X buffer as previously described (11). After overnight incubation at 4°C on a rotating device, immune complexes were precipitated at 4°C for only 2 h on a rotating device with 35 μL of protein A/G magnetic beads. Immunoprecipitated (IP) complexes were then washed four times with 750 μL of RIPA 1X buffer before denaturation in Laemmli buffer at RT for western blot analysis.

#### Western blot

Soluble proteins (10–50 μg) were analyzed on 12% SDS-PAGE gels. Proteins were transferred to nitrocellulose membranes and blocked for 1 h in Tris-buffered saline with 0.1% [v/v] Tween-20 (TBS-T) containing 5% [w/v] skim milk or BSA with constant shaking. Membranes were then incubated with primary antibodies diluted in TBS-T with 5% skim milk or BSA overnight at 4°C with constant shaking. The blots were then washed with TBS-T and incubated at RT with horseradish peroxidase labeled secondary antibodies diluted in 5% skim milk or BSA/TBS-T for 1 h. Following the secondary incubation, the membranes were washed with TBS-T before blots imaging. Equal protein loading was confirmed using GAPDH and sarcomeric actin immunoblotting.

For western blots of desmin-IP proteins, membranes were first incubated with RL2 antibody diluted in 5% fresh BSA overnight at 4°C before incubation with other antibodies.

#### Phos-tag™ gels

Soluble proteins were analyzed on 10% gels containing 40 μM of Phos-tag™ (Wako, Osaka, Japan) and 100 μM of Mn^2+^ at 90V for 2.5 h. The excess of metal was removed by washing the gels three times for 10 min in transfer buffer (NuPAGE® Transfer Buffer, Invitrogen) containing 10% methanol and 10 mM EDTA and then three times for 10 min in transfer buffer containing 10% methanol before the proteins were transferred onto 0.2 μm PVDF membrane. To detect desmin and its phosphorylated forms, membranes were blocked 1 h in 5% skim milk/TBS-T before overnight incubation at 4°C with desmin antibody diluted 1/1,000 in blocking solution. The following steps were similar to those described above for western blot. Briefly, the Phos-tag™ molecules incorporated into the SDS-PAGE are able with the cooperation of two Mn^2+^ metal cations to slow down the migration of phosphorylated proteins. Therefore, phosphorylated desmin migrates at higher apparent molecular weight than the non-phosphorylated form. However, at equivalent phosphorylation levels, the position of the phosphate group can also influence the apparent molecular weight of a protein in a Phos-tag™ gel.

#### WGA

Soluble proteins (50 μg) were analyzed on 7% gels containing 3.75 mg/mL of Wheat Germ Agglutinin (WGA) (L9640, Sigma-Aldrich) at 4°C at 20 mA for 2 h as previously described (15). After migration, the same protocol for protein transfer and incubation with primary and secondary antibodies as for classical western blot was applied.

#### List of antibodies

**Table:** 

**Protein**	**Sample**	**Reference company**	**Dilution antibodies**
Desmin	Heart	ab32362 Abcam	1/1,000
	NCM		1/5,000
O-GlcNac	Heart/NCM	NB300-524 (RL2) Novus Biological	1/2,000
OGT	NCM	clone DM17, O6264 Sigma-Aldrich	1/1,000
GAPDH	Heart/NCM	sc-36562 Santa Cruz	1/5,000
Phospho-serine	Heart/NCM	P5747, Sigma-Aldrich	1/1,000
Sarcomeric actin	Heart /NCM	MO874	1/2,000
		Dako	

#### Blots imaging

The Chemidoc® XRS+ camera (Biorad) and the Image Lab™ software were used for blots imaging and densitometry analysis. Membranes were incubated for 5 min with Clarity™ Western ECL Substrate (Biorad) before imaging. The signal was quantified from the image obtained just before saturation. The band corresponding to the protein of interest was framed within a defined area to express the signal intensity depending of the area. This value was related to the intensity value of the reference protein (GAPDH or sarcomeric actin). The values were expressed in arbitrary units (U.A.).

### Statistical analysis

Data expressed as means ± SEM were analyzed with GraphPad Prism version 6.01 (GraphPad Software, San Diego, CA) and comparisons were made by Student's *t*-test, Mann-Whitney (two-tailed), one- or two-way analysis of variance (ANOVA) with Tukey's *post-hoc* test, as appropriate. Results were considered statistically significant if the *p* < 0.05.

## Results

### Post-translational modification of desmin heart failure rats

**Figure 1:**
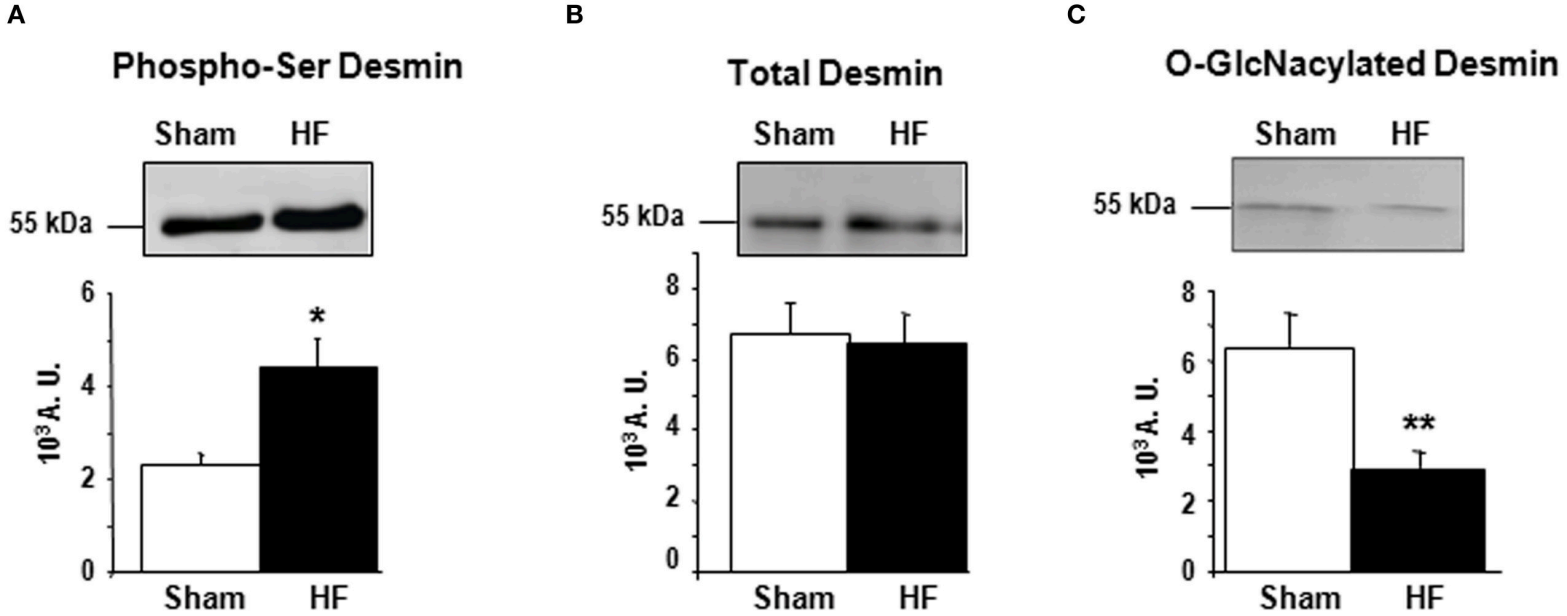
Post-translational modifications of desmin of LV from HF-rats. Quantification of Ser-phosphorylated desmin **(A)**, total desmin levels **(B)**, and O-GlcNAcylated desmin levels **(C)** in LV (50 μg) of sham- (*n* = 11) (white box) and HF- (*n* = 11) (black box) rats at 2 months after MI. The positions of molecular weight are indicated as kilodalton (kDa) on the left. An internal standard was loaded in each gel for the standardization and quantification. Graphs show mean ± SEM values expressed in arbitrary units (A.U.). *P < 0.05; **P < 0.01.

Cardiac remodeling and dysfunction in HF-rats was characterized at 2 months post-MI by significant increases in LV end-diastolic pressure, LV end-diastolic and end-systolic diameters, and LV weight as previously shown (10). We previously investigated cardiac phosphoproteomic changes associated with LV remodeling and dysfunction in this HF-rat model. At 2 months after surgery, proteomic analysis revealed different LV phosphoproteomic patterns between the sham- and HF-rats (16). We previously identified two spots as being desmin (11). We highlighted a significant increase of desmin phospho-species in LV of rats 2 months after MI compared to controls (Figure 1A) without any modulation of total desmin protein levels (Figure 1B). Then, we looked for interplay between phosphorylation and O-GlcNAcylation of desmin in the same experimental model and found a significant decrease of O-GlcNAcylated desmin in LV of HF rats compared to controls (Figure 1C). These data showed that desmin may bear O-GlcNAc residues and that the levels of O-GlcNAcylated desmin were inversely related to the levels of phosphorylated desmin in LV of HF rats. To confirm these data, we tested several modulators of O-GlcNAcylation in several *in vitro* and *ex vivo* models, such as primary culture of cardiomyocyte (NCM) and isolated perfused heart.

### Modulation of O-GlcNAcylation *in vitro* in primary cultures of neonate cardiomycytes

#### Inhibition of OGA by thiamet G

**Figure 2:**
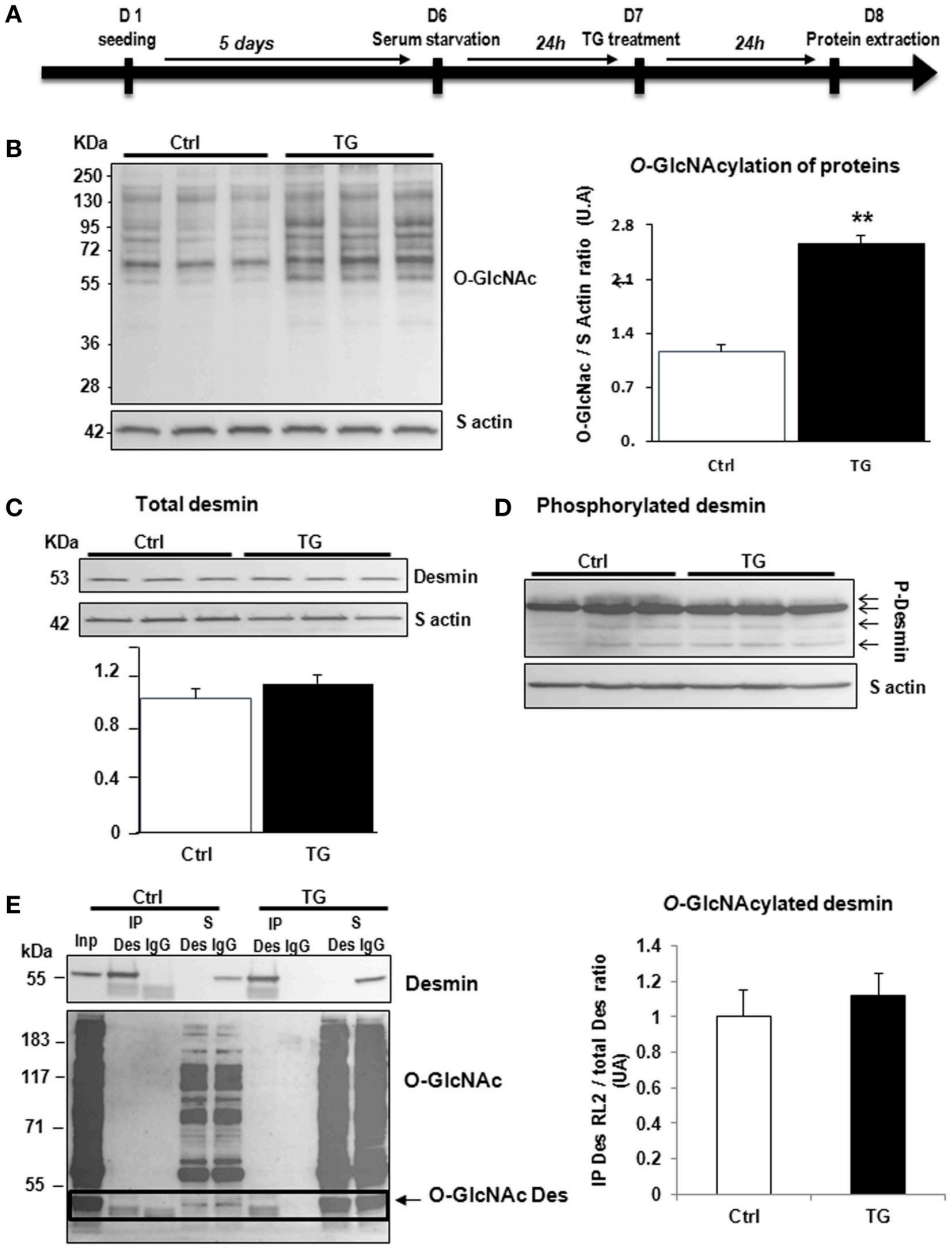
Impact of OGA inhibition by thiamet G in primary cultures of NCM. **(A)** Description of the protocol designed for Thiamet G (TG) treatment of primary culture of neonate cardiomyocytes (NCM). **(B)** Representative western blots (left panel) and quantification (right panel) of O-GlcNAcylated proteins levels in control (Ctrl) and NCM treated with 100 nM of thiamet G (TG) during 24 h (*n* = 12). **(C)** Western blots (upper panel) and quantification (lower panel) of total desmin levels in the same samples. **(D)** Phosphorylation profiles of desmin were analyzed in the same samples by Phos-tag™ gel. **(E)** Representative immunoprecipation (IP) with desmin antibody before western blot with RL2 antibody (left panel) and quantification (right panel) of *O*-GlcNAcylated desmin levels in the same samples. Inp, input; IP, immunoprecipitated proteins; S, IP supernatant; Des, desmin; IgG, Immunoglobulin. The arrow indicates the *O*-GlcNAcylated desmin. Data are expressed as means of an arbitrary unit (A.U.) ± SEM. The positions of molecular weight are indicated as kilodalton (kDa) on the left. **P < 0.01.

First, we studied the impact of OGA inhibition by thiamet G in primary cultures of neonate rat cardiomyocytes (NCM) as designed (Figure 2A) and observed a significant increase of O-GlcNAcylated proteins (1.16 ± 0.09 vs. 2.55 ± 0.27, *P* < 0.01) (Figure 2B). Thiamet G treatment has no effect on total desmin levels (Figure 2C) nor on desmin phosphospecies (Figure 2D). To quantify the levels of O-GlcNAcylated desmin, we used immunoprecipitation (IP) of desmin species followed by a western blot with RL2 antibody to detect O-GlcNAcylated proteins (Figure 2E). We verified by desmin western blot the efficiency of IP with desmin detected in input, IP (IP Des) and supernatant of IgG IP (S igG) but not in supernatant of desmin IP (S Des). The specificity of this approach was also verified with no O-GlcNAcylated proteins detected in beads. As observed the levels of O-GlcNAcylated desmin was very low in NCM and we did not observe any modulation in NCM treated with thiamet G (Figure 2E).

#### Silencing of OGT

**Figure 3:**
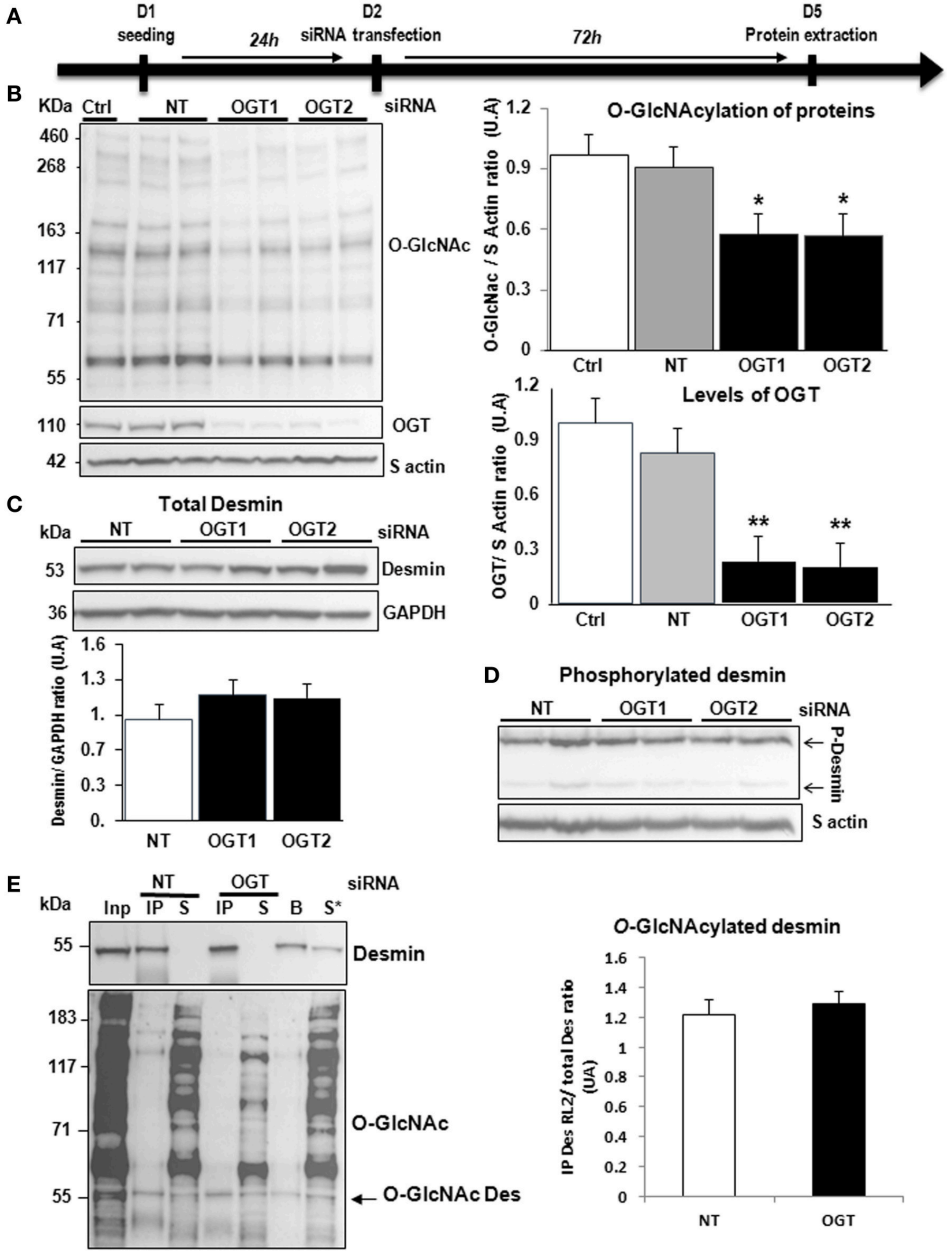
Effect of OGT silencing in primary cultures of NCM. **(A)** Description of the protocol designed for OGT silencing of primary cultures of NCM. **(B)** Western blots (left panel) and quantification of O-GlcNAcylated proteins levels (upper and right panel) and OGT (lower and right panel) in primary cultures of NCM transfected with non-targeting (NT) siRNA control and OGT1 and OGT2 siRNA (*n* = 12). **(C)** Western blots (upper panel) and quantification (lower panel) of total desmin levels in the same samples. **(D)** Phosphorylation profiles of desmin was analyzed in the same samples by Phos-tag™ gel. **(E)** Representative Immunoprecipitation (IP) with desmin antibody before western blot with RL2 antibody (left panel) and quantification (right panel) of O-GlcNAcylated desmin levels in the same samples. Inp, input; IP, immunoprecipitated proteins; S, IP supernatant; B, beads alone; S*, IP supernatant of beads. The arrow indicates the O-GlcNAcylated desmin. Graphs show mean ± SEM values expressed in arbitrary units (A.U.). The positions of molecular weight are indicated as kilodalton (kDa) on the left. *P < 0.005; **P < 0.01.

Second, we studied the impact of OGT silencing in primary cultures of neonate rat cardiomyocytes (NCM) as designed (Figure 3A) and observed a significant decrease of O-GlcNAcylated proteins [1.08 ± 0.03 vs. 0.54 ± 0.08 (OGT1) and 0.52 ± 0.07 (OGT2), *P* < 0.05] that is due to the significant decrease of OGT validating the efficiency of OGT silencing [0.99 ± 0.13 vs. 0.23 ± 0.02 (OGT1) and 0.20 ± 0.05 (OGT2), *P* < 0.01] (Figure 3B). Both OGT siRNA1 and siRNA 2 did not show any effect on total desmin levels (Figure 3C) nor in desmin phosphospecies (Figure 3D). We then quantified the levels of O-GlcNAcylated desmin as described in Figure 2E. Conversely, we observed an unspecific low intensity band for O-GlcNAcylated desmin with beads but we did not observe any modulation of O-GlcNAcylated desmin after OGT silencing in NCM (Figure 3E).

### Modulation of O-GlcNAcylation *ex vivo* in isolated perfused heart

**Figure 4:**
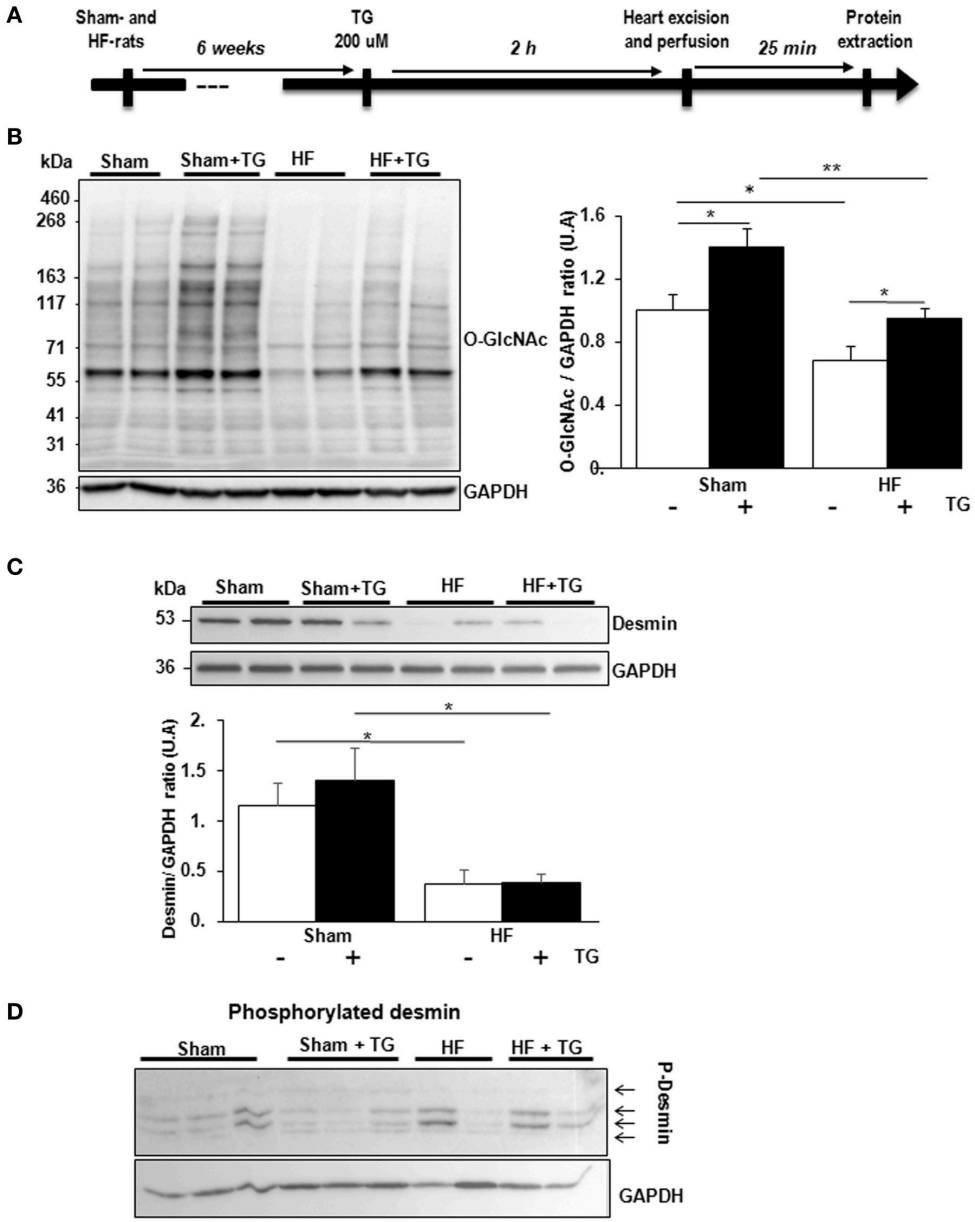
Effect of OGA inhibition by thiamet G in isolated perfused heart. **(A)** Description of the protocol designed for thiamet G (TG) perfusion in sham- (*n* = 6) and HF- (*n* = 7) rats 6 weeks post-MI. **(B)** Western blot (left panel) and quantification (right panel) of *O*-GlcNAcylated proteins levels measured in proteins extracted from LVs of isolated perfused sham- and HF-rat hearts treated or not with 100 μM thiamet G for 2 h (*n* = 7 in each group). **(C)** Western blots (upper panel) and quantification (lower panel) of total desmin levels in the same samples. **(D)** Phosphorylation profiles of desmin were analyzed in the same samples by Phos-tag™ gel. Graphs show mean ± SEM values expressed in arbitrary units (A.U.). The positions of molecular weight are indicated as kilodalton (kDa) on the left. *P < 0.05; ** < 0.01.

In isolated perfused hearts, we assessed the functional cardiac modification induced by thiamet G, a specific OGA inhibitor, injected 2 h before the perfusion (Figure 4A). Thiamet G decreased significantly the coronary flow in only sham-rats. In HF-rats, thiamet G increased significantly LV developed pressure and cardiac output without any effect on heart rate (Table 1).

**Table 1:** Modulation of OGA inhibition by thiamet G in isolated perfused heart.

**Parameters**	**Sham-rats**		**HF-rats**	
	**Ctrl (*****n*** = **6)**	**TG (*****n*** = **6)**	**Ctrl (*****n*** = **6)**	**TG (*****n*** = **7)**
LV developed pressure (mmHg)	134 ± 2	135 ± 10	84 ± 11	99 ± 10^*^
Coronary flow (mL/min)	29 ± 2	21 ± 6^*^	28 ± 4	34 ± 5
Heart rate (bpm)	248 ± 1	243 ± 12	316 ± 24	283 ± 14
Cardiac output (mmHg)	48 ± 5	47 ± 9	30 ± 2	33 ± 2^*^

*Ctrl, perfusion without pharmacological agents; TG, thiamet G (200 μM); bpm, beats per minute*.

* *P < 0.05 vs. control in each group*.

We validated by western blot the inhibitory effect of OGA by thiamet G with significant increase of O-GlcNAcylated LV proteins in sham- and HF-rat heart perfused with the inhibitor (Figure 4B). We found a significant decrease of total desmin expression in LV from HF-rat perfused compared to the sham-rats independently of thiamet G perfusion (Figure 4C). We visualized desmin phosphospecies by desmin immunoblot of Phos-tag™ gels and we did not find any modulation with different molecular weight desmin species detected in thiamet G-perfused heart (Figure 4D).

### New technology to detect O-GlcNAcylated proteins

**Figure 5:**
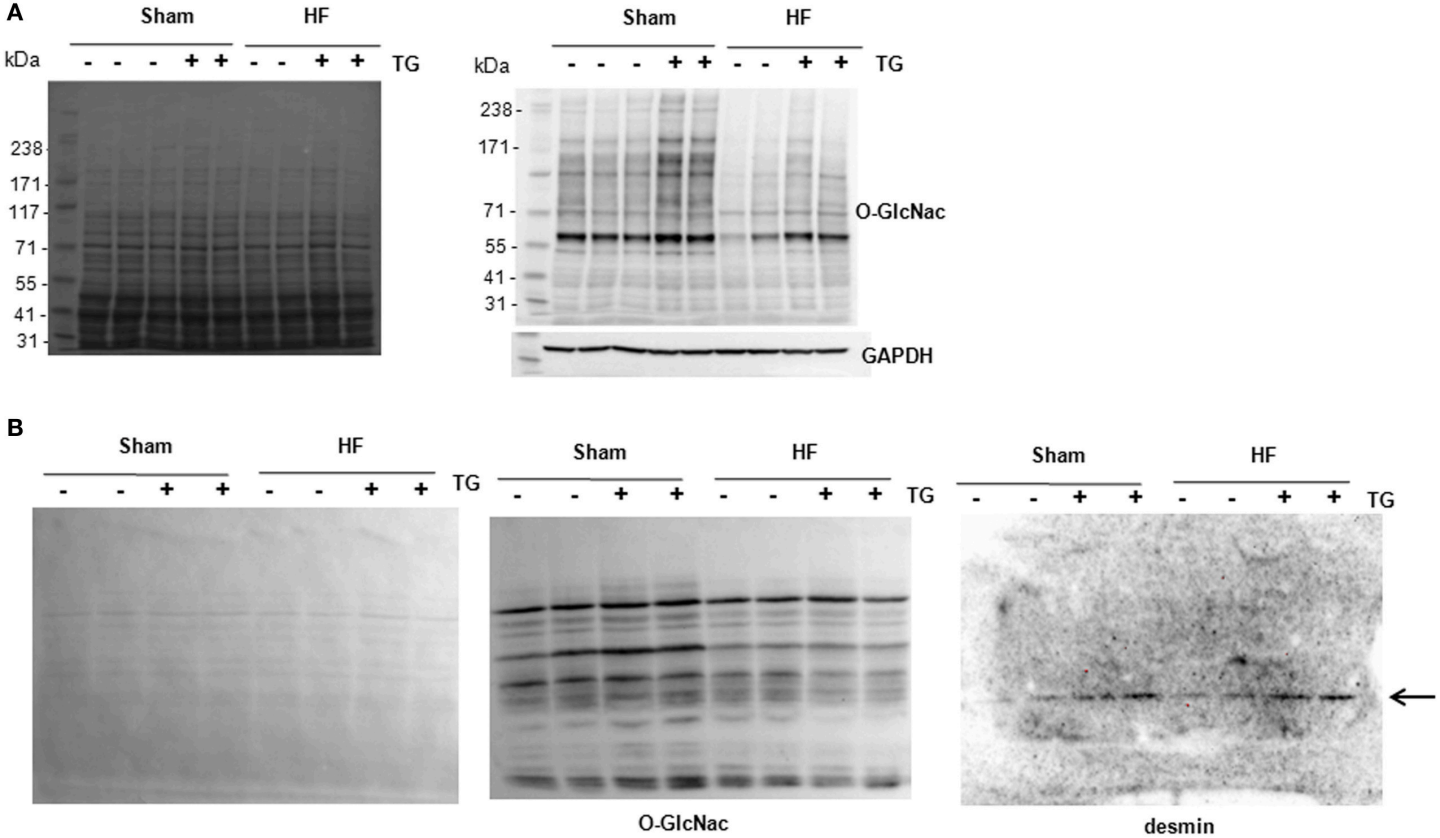
Analysis of O-GlcNAcylated LV proteins by Western blot and WGA-SDS-PAGE gel electrophoresis. **(A)** Red ponceau staining (left panel) and western blot (right panel) of O-GlcNAcylated proteins (50 μg) extracted from sham- and HF-rats treated or not with thiamet G. The positions of molecular weight are indicated as kilodalton (kDa) on the left. **(B)** Red ponceau staining (left panel) and WGA-SDS-PAGE of O-GlcNAcylated proteins levels (middle panel) of O-GlcNAcylated desmin levels (right panel) from the same samples. The arrow in desmin WGA gels indicates the non-O-GlcNAcylated form.

Due to the difficulties to detect specifically *O*-GlcNAcylated desmin in LV proteins either from cultures of cardiomyocytes or perfused heart, we compare the sensitivity of 2 methods, western blots of O-GlcNAcylated LV proteins separated with classical SDS-PAGE gel (Figure 5A) and WGA-SDS-PAGE gel (Figure 5B) as recently described (15). First, red ponceau staining of the transferred membranes showed a less sensitivity to detect protein profiles with WGA gel. But conversely, we observed for the detection of *O*-GlcNAcylated LV proteins with RL2 antibody a stronger signal of better quality with WGA gel (Figure 5B) by comparison to SDS gel (Figure 5A). We then tested the detection of desmin in WGA gel and only found one band with a stronger signal in thiamet G treated samples either in sham- or HF-rats (Figure 5B) that we were unable to quantify due to the strong background.

## Discussion

Post-translational modifications of sarcomeric proteins play an important role in HF-induced cardiac dysfunction (17, 18). We previously found a 2-fold increased phosphorylation of desmin levels by phosphoproteomic analysis (11) in a well characterized rat ischemic HF experimental model in which the induction of anterior MI leads to LV remodeling and to HF (19). Nine serine resides conserved between species were identified by mass spectrometry to be phosphorylated (11). In the present study, we found a decrease of O-GlcNAcylation levels of desmin and we hypothesize that a cross-talk between phosphorylation and O-GlcNAcylation of desmin may occur during HF development as we have recently shown for troponin T in in the same rodent model of HF (10). Here, our aim was to determine whether the levels of O-GlcNAcylated desmin are regulated in cardiomyocytes by inhibition or activation of O-GlcNAcylation and whether modulation of O-GlcNAcylation impacts the phosphorylation levels of desmin, in order to identify the serine residues that could be involved in the interplay.

For that purpose, first, we have modulated the levels of O-GlcNAcylated proteins in primary culture of neonate rat cardiomyocytes by either acting on OGA inhibition by treatment of the cardiomyocytes with a specific OGA inhibitor, thiamet G (20), or by OGT silencing. We verified that both treatments were efficient in primary cultures of cardiomyocytes with significant increase of O-GlcNAcylated proteins after OGA inhibition and significant decrease of O-GlcNAcylated proteins after OGT silencing. With both treatments, we did not observe any changes in total desmin protein levels and in the expression of desmin PTMs: phosphorylation and O-GlcNAcylation.

Second, we used an *ex vivo* model of perfused rat heart in order to test the efficiency of thiamet G on cardiac contractility. We found a positive impact of OGA inhibition on several cardiac parameters which were different depending on the sham- (coronary flow) or HF- (LV developed pressure and cardiac output) animals, except for the heart rate which was not modified in both groups of animals after a 2 h perfusion of thiamet G. We verified the efficiency of OGA inhibition by the significant increase of O-GlcNAcylated proteins in perfused heart in both group of animals treated (1.4-fold, *P* < 0.05). And as shown in *in vitro* experiments, we did not observe any modulation of total desmin and its phosphorylated forms, except a significant decrease of desmin expression between sham- and HF- rats after the 2 h perfusion, that can be explained by a degradation during the perfusion, such as calpain (11). We found these negative results by 2 techniques: IP desmin followed by phospho-serine WB (not shown) and Phos-tag™ gels.

Despite these negative results with *in vitro* and *ex vivo* cardiac models of O-GlcNAcylation modulation, we did not want to exclude that the technology used to quantify the levels of O-GlcNAcylated desmin was inappropriate or not enough sensitive. To clarify this hypothesis, we have tested a technique recently published (15). Despite the less sensitivity of the technique to detect the proteins transferred into the membrane (red ponceau staining), we improved the quality of the profile of O-GlcNAcylated proteins by comparison to a classical western blot with the use of the same antibody against the O-GlcNAc moieties. Unfortunately, the quality of the WGA-desmin blot did not allow quantifying the desmin band detected despite the highest intensity of desmin in thiamet G-treated LV samples. Further improvement will be required to use this promising technique to quantify the levels of O-GlcNAcylation of a specific protein.

Our data have shown that despite the presence of O-GlcNAc moieties on desmin from heart, there is no regulation of O-GlcNAcylated desmin by modulators of O-GlcNAcylation and subsequently there is no interplay between phosphorylation and O-GlcNAcylation of desmin.

The future is to find the targets of O-GlcNAcylation in heart that can be involved in cardiac improvement and to find the pharmacological agents able to decrease the levels of phosphorylated desmin which is toxic due to the aggregation of phosphorylated desmin in failing heart.

## Data availability

The data, analytic methods, and study materials presented in this study will be been made available to other researchers for purposes of reproducing the results or replicating the procedure.

## Author contributions

MB and ED-D designed the study, make experiments and wrote the paper. TM, AD, and OB make experiments. VR, PM, and FP designed the study and wrote the paper.

### Conflict of interest statement

The authors declare that the research was conducted in the absence of any commercial or financial relationships that could be construed as a potential conflict of interest.

## References

[1] Pfeffer M Braunwald E. Ventricular remodeling after myocardial infarction. Experimental observations and clinical implications. Circulation (1990) 81:1161–72. 10.1161/01.CIR.81.4.1161 2138525

[2] Savoye C Equine O Tricot O Nugue O Segrestin B Sautière K. Left ventricular remodeling after anterior wall acute myocardial infarction in modern clinical practice (from the REmodelage VEntriculaire [REVE] Study Group). Am J Cardiol. (2006) 98:1144–9. 10.1016/j.amjcard.2006.06.011 17056315

[3] Fertin M Hennache B Hamon M Ennezat PV Biausque F Elkohen M. Usefulness of serial assessment of B-type natriuretic peptide, troponin I, and C-reactive protein to predict left ventricular remodeling after acute myocardial infarction (from the REVE-2 Study). Am J Cardiol. (2010) 106:1410–6. 10.1016/j.amjcard.2010.06.071 21059429

[4] Bauters C Dubois E Porouchani S Saloux E Fertin M de Groote P. Long-term prognostic impact of left ventricular remodeling after a first myocardial infarction in modern clinical practice. PLoS ONE (2017) 12:e0188884. 10.1371/journal.pone.0188884 29176897 PMC5703528

[5] Hamdani N Kooij V Van Dijk S Merkus D Paulus WJ Remedios CD. Sarcomeric dysfunction in heart failure. Cardiovasc Res. (2008) 77:649–58. 10.1093/cvr/cvm079 18055579

[6] Zachara NE. The roles of O-linked β-N-acetylglucosamine in cardiovascular physiology and disease. AJP Hear Circ Physiol. (2012) 302:H1905–18. 10.1152/ajpheart.00445.2011 22287582 PMC3362101

[7] Wang Z Gucek M Hart GW. Cross-talk between GlcNAcylation and phosphorylation: site-specific phosphorylation dynamics in response to globally elevated O-GlcNAc. Proc Natl Acad Sci USA. (2008) 105:13793–8. 10.1073/pnas.0806216105 18779572 PMC2544533

[8] Ramirez-Correa GA Jin W Wang Z Zhong X Gao WD Dias WB. O-linked GlcNAc modification of cardiac myofilament proteins: a novel regulator of myocardial contractile function. Circ Res. (2008) 103:1354–8. 10.1161/CIRCRESAHA.108.184978 18988896 PMC2615199

[9] Hu P Shimoji S Hart GW. Site-specific interplay between O-GlcNAcylation and phosphorylation in cellular regulation. FEBS Lett. (2010) 584:2526–38. 10.1016/j.febslet.2010.04.044 20417205

[10] Dubois-Deruy E Belliard A Mulder P Bouvet M Smet-Nocca C Janel S. Interplay between troponin T phosphorylation and O-N-acetylglucosaminylation in ischaemic heart failure. Cardiovasc Res. (2015) 107:56–65. 10.1093/cvr/cvv136 25916824

[11] Bouvet M Dubois-Deruy E Alayi TD Mulder P El Amranii M Beseme O. Increased level of phosphorylated desmin and its degradation products in heart failure. Biochem Biophys Rep. (2016) 6:54–62. 10.1016/j.bbrep.2016.02.014 28955862 PMC5600436

[12] Lowery J Kuczmarski ER Herrmann H Goldman RD. Intermediate filaments play a pivotal role in regulating cell architecture and function. J Biol Chem. (2015) 290:17145–53. 10.1074/jbc.R115.640359 25957409 PMC4498054

[13] Mulder P Devaux B Richard V Henry J Wimart M Thibout E. Early versus delayed angiotensin-converting enzyme inhibition in experimental chronic heart failure. Effects on survival, hemodynamics, and cardiovascular remodeling. Circulation (1997) 95:1314–9. 10.1161/01.cir.95.5.1314 9054865

[14] Cieniewski-Bernard C Mulder P Henry JP Drobecq H Dubois E Pottiez G. Proteomic analysis of left ventricular remodeling in an experimental model of heart failure. J Proteome Res. (2008) 7:5004–16. 10.1021/pr800409u 18922030

[15] Kubota Y Fujioka K Takekawa M. WGA-based lectin affinity gel electrophoresis: a novel method for the detection of O-GlcNAc-modified proteins. PLoS ONE (2017) 12:1–12. 10.1371/journal.pone.0180714 28686627 PMC5501588

[16] Dubois-Deruy E Belliard A Mulder P Chwastyniak M Beseme O Henry J-P. Circulating plasma serine^208^-phosphorylated troponin T levels are indicator of cardiac dysfunction. J Cell Mol Med. (2013) 17:1335–44. 10.1111/jcmm.12112 23905701 PMC4159027

[17] Wu SC Solaro RJ. Protein kinase C: a novel regulator of both phosphorylation and de-phosphorylation of cardiac sarcomeric proteins. J Biol Chem. (2007) 282:30691–8. 10.1074/jbc.M703670200 17724026 PMC2597085

[18] Dubois E Richard V Mulder P Lamblin N Drobecq H Henry J-P. Decreased serine207 phosphorylation of troponin T as a biomarker for left ventricular remodelling after myocardial infarction. Eur Heart J. (2011) 32:115–23. 10.1093/eurheartj/ehq108 20418543

[19] Mulder P Barbier S Chagraoui A Richard V Henry JP Lallemand F. Long-term heart rate reduction induced by the selective I(f) current inhibitor ivabradine improves left ventricular function and intrinsic myocardial structure in congestive heart failure. Circulation (2004) 109:1674–9. 10.1161/01.CIR.0000118464.48959.1C 14981003

[20] Vocadlo DJ. O-GlcNAc processing enzymes: catalytic mechanisms, substrate specificity, and enzyme regulation. Curr Opin Chem Biol. (2012) 16:488–97. 10.1016/j.cbpa.2012.10.021 23146438

